# The association between leukocytes and sperm quality is concentration dependent

**DOI:** 10.1186/1477-7827-8-12

**Published:** 2010-02-05

**Authors:** Jakob E Lackner, Ashok Agarwal, Reda Mahfouz, Stefan S du Plessis, Georg Schatzl

**Affiliations:** 1 Department of Urology, KH Hietzing mit Neurologischem Zentrum Rosenhügel & Ludwig Boltzmann Institut für Andrologie und Urologie, Vienna, Austria; 2 Center for Reproductive Medicine, Glickman Urological and Kidney Institute, Glickman Tower, Cleveland Clinic, Cleveland, Ohio, USA; 3 Division of Medical Physiology, University of Stellenbosch, South Africa; 4 Department of Urology, Medical University of Vienna, Vienna, Austria

## Abstract

**Background:**

To evaluate the association between leukocytes (polymorphonuclear granulocytes -PMNL) and semen parameters at different leukocyte concentrations.

**Methods:**

This was a retrospective clinical study at a university hospital andrology clinic. Semen samples from infertile men were analyzed for sperm morphology and motility according to seminal leukocytes (PMNL) concentration (category A: >0 to <0.25 × 10(6)/mL; category B: >0.25 to <0.5 × 10(6)/mL; category C: >0.5 to <0.75 × 10(6)/mL; category D: >0.75 to <1.0 × 10(6)/mL, category E: >1 × 10(6)/mL).

**Results:**

The percentage of sperm with normal morphology increased significantly from category A (14%) to category D (19%) but decreased in category E to levels (14%) similar to those in category A. Motility grades a and a+b (combined) also increased from category A (12%, 20%) to category D (18.0%, 28.5%) and decreased in category E (11%, 20.5%) to levels similar to those in category A. Sperm deformities and motility grades c and d increased progressively in all categories.

**Summary:**

Leukocytes had a positive association with normal morphology and progressive motility in semen samples at a concentration of 0-1 × 10(6)/mL. The findings suggest that the association between leukocytes (PMNL) and semen quality might be concentration dependent.

## Background

The association between seminal leukocytes and semen quality is still a matter of debate in the literature. Leukocytospermia, defined by the World Health Organization (WHO) as more than 1 million leukocytes per millilitre, has an incidence of 10-20% in the general population and is especially common in infertile men. However, lower concentrations of seminal leukocytes (0-1 × 10^6^/mL) are still more widespread, and are seen even in the absence of infection [1-6].

As leukocytospermia is so prevalent in infertile men, it can be questioned as to whether the presence of seminal leukocytes correlates with semen quality. Early studies found leukocytes to have a positive effect on semen quality [7,8]. Tomlinson et al. reported that leukocytes phagocytosed abnormal spermatozoa [7], whereas Kiessling et al. found an improvement in sperm motility in semen samples with a leukocyte concentration of >2 × 10^6^/mL [8]. However, the results of more recent studies suggest that leukocytes negatively impact on semen quality as a result of the presence of reactive oxygen species (ROS), which are primarily produced by leukocytes. It is believed that ROS are harmful to spermatozoa [9-13]. Aziz et al. reported a positive correlation between leukocytospermia and sperm tail defects, acrosomal damage and high sperm deformity index scores [13]. However, in another study, Ziyyat et al. [14] reported an increase in sperm motility in semen samples with moderate leukocytospermia (defined as seminal leukocytes <1 × 10^6^/mL), but observed a paradoxical decrease in sperm motility in semen samples exceeding a threshold of 1 × 10^6 ^leukocytes/mL. Similar results were found for semen samples classified as having normal morphology (sperm deformities were not described in this study). In support of this finding, Lackner et al. have shown that leukocytospermia may not necessarily have a negative impact on outcomes following assisted reproductive techniques. They reported similar fertilization rates for non-leukocytospermic samples and leukocytospermic samples (63.4% vs. 64.3%, *P *= not significant) [15]. Corresponding pregnancy rates also did not differ significantly between the two groups.

Considering the results from these reports, it is possible that leukocytes in semen samples may have a dual effect on semen parameters. The aim of this study was therefore to evaluate the association between different concentrations of seminal leukocytes and various semen parameters in infertile men. Infertile men were chosen for this study, as these men usually have a higher prevalence of leukocytospermia than healthy males.

## Methods

Males attending clinics for fertility examination in the Department of Urology at the Medical University of Vienna who were found to be infertile (defined as an abnormal semen analysis, according to WHO criteria [1]) were invited to participate in this study. A detailed clinical urological examination was performed, including examination of the testicles, scrotum and penis, with the aim of ruling out a possible male accessory gland infection, as recommended by the WHO. Men with acute urogenital infections and testicular tumors were also excluded from the study, as were men with a general clinical infection 1 week prior to the semen analysis.

All semen analyses were performed by the same highly trained laboratory technologist within 1 hour after ejaculation, and analyzed according to the criteria as specified in the WHO Laboratory Manual [1]. Data on sperm and leukocyte concentrations, and patient age were collected. Microscopic analysis of sperm concentration and sperm motility was performed by means of computer-aided semen analysis (CASA) using a Hamilton Thorne motility analyzer (05028 74; Hamilton Thorne Research Inc., Danvers, MA, USA) with 400× magnification. Sperm motility was graded according to WHO criteria as follows: grade a - fast progressive, grade b - slow progressive, grade c - non-progressive and grade d - immotile.

Prestained morphology slides (Testsimplets; Waldeck GmbH, Münster, Germany) were used to study sperm morphology. One drop of the semen sample was placed on the Testsimplets slide, incubated at room temperature for 2 hours and examined under 600× magnification. Morphology was evaluated according to WHO criteria [1] using a threshold value for normal spermatozoa of 30%. Besides the normal forms observed, all head, midpiece, and tail defects were also recorded.

Leukocyte concentration was determined after special peroxidase staining and counting under 600× magnification, as described by Endtz [16]. Thus, polymorph nuclear granulocytes, which are the most prevalent cell type in semen, were stained [2]. Therefore we used the term leukocytes as synonym for polymorphonuclear granulocytes, as described by the WHO laboratory manual 1999 [1].

To study the association of different leukocyte concentrations on semen parameters, samples were categorized as follows:

• category A: leukocyte concentration >0 to <0.25 × 10^6^/mL

• category B: leukocyte concentration >0.25 to <0.5 × 10^6^/mL

• category C: leukocyte concentration >0.5 to <0.75 × 10^6^/mL

• category D: leukocyte concentration >0.75 <1.0 × 10^6^/mL

• category E: leukocyte concentration >1 × 10^6^/mL.

The Statistical Package for the Social Sciences (SPSS 10.0.7, SPSS. Inc. 1989-1999) computer software program was used for all statistical analysis. Because of the distribution of the data, all demographic data are presented as the median and 25th and 75th quartiles. Correlations between leukocyte concentrations and semen parameters were analyzed using the Spearman Rho test. An *r *value of >0.1 was seen for all semen parameters, indicating at least a weak correlation (suggesting borderline clinical significance) with leukocyte concentration. Semen samples were then assigned to a leukocyte category, as described above. A linear regression model was used to show the trends for the different semen parameters within the leukocyte categories. Statistical differences were assessed with the use of the Mann-Whitney test. A *P *value < 0.05 was defined as statistically significant.

## Results

A total of 618 semen analyses were included for further evaluation. The median overall age of donors was 35.0 years (range, 29.0-40.0 years).

The median sperm concentration in this study population was 4.05 × 10^6^/mL (range, 0.875-8.70 × 10^6^/mL), a median 16.5% of sperm had normal morphology (range, 6.0-29.0%), 28.0% (range, 14.0-38.0%) had head deformities, 28.0% (range, 14.0-38.0%) had midpiece deformities, and 7.0% (range, 2.0-11.0%) had tail deformities.

With regard to motility, 16.0% of sperm were assigned to grade a (range, 0.0-35.25%), 7.0% were grade b (range, 0.0-14.0%), 4.0% were grade c (range, 0.0-9.0%) and 50.0% were grade d (range, 22.0-79.0%). The median leukocyte concentration of all 618 semen samples was 0. 25 × 10^6^/mL (range, 0.0-0.5 × 10^6^/mL).

A total of 269 samples were assigned to leukocyte concentration category A, 140 samples to category B, 97 samples to category C, 52 samples to category D and 60 samples to category E. For detailed results of semen analysis according to category, see Table 1. Table 1 also presents the statistical differences between the leukocyte categories using category A (with the lowest leukocyte concentration) as the referent.

**Table 1:** Sperm parameters according to leukocyte concentration category

	Leukocyte concentration category					Statistical comparisons between groups^a^			
	A	B	C	D	E	A/B	A/C	A/D	A/E
Age (years)	35.0 (29.0-40.0)	35.0 (30.0-41.0)	36.0 (31.0-41.0)	33.5 (29.0-40.0)	33.0 (27.0-39.75)	0.629	0.176	0.762	0.14
Sperm concentration (10^6^/mL)	2.6 (0.1-7.25)	5.5 (2.2-11.76)	4.6 (1.9-8.7)	4.6 (1.8-9.76)	3.6 (1.6-9.53)	**0.001**	**0.001**	**0.002**	**0.014**
Normal morphology (%)	14.0 (0-26.5)	19.0 (11.25-32.0)	18.0 (11.0-37.0)	19.0 (11.25-26.75)	14.0 (7.25-25.75)	**0.001**	**0.003**	**0.021**	0.452
Head deformities (%)	24.0 (0.0-35.0)	31.0 (18.25-39.0)	32.0 (22.5-39.0)	31.5 (22.5-40.0)	31.0 (20.0-39.0)	**0.001**	**0.001**	**0.001**	**0.004**
Midpiece deformities (%)	22.0 (0.0-35.0)	29.0 (20.0-36.0)	30.0 (20.0-39.0)	32.5 (22.5-38.0)	31.5 (20.5-39.0)	**0.001**	**0.001**	**0.001**	**0.001**
Tail deformities (%)	5.0 (0.0-11.0)	7.0 (4.0-11.0)	7.0 (3.0-10.5)	9.0 (6.0-12.0)	8.0 (4.0-12.0)	**0.004**	**0.041**	**0.001**	**0.016**
Motility grade a+b (%)	20.0 (0-45-0)	32.0 (9.0-60.75)	29.0 (10.0-46.5)	28.5 (8.25-56.75)	20.5 (10.0-39.25)	**0.001**	0.123	0.09	0.588
Motility grade a (%)	12.0 (0.0-31.0)	22.5 (4.25-44.0)	19.0 (3.0-36.0)	18.0 (4.25-36.75)	11.0 (0.5-27.0)	**0.001**	0.216	0.086	0.989
Motility grade b (%)	5.0 (0.0-13.0)	9.0 (3.0-15.0)	8.0 (0.0-15.0)	9.0 (0.0-15.0)	7.0 (0.0-13.0)	**0.001**	**0.04**	0.076	0.29
Motility grade c (%)	2.0 (0.0-8.0)	5.0 (2.0-10.0)	5.0 (0.5-11.0)	4.5 (0.25-8.0)	5.0 (0.0-10.0)	**0.001**	**0.002**	0.072	**0.026**
Motility grade d (%)	42.0 (1.5-76.0)	51.0 (23.2-73.5)	56.0 (31.0-87.0)	56.0 (31.0-87.0)	68.0 (35.5-85.0)	**0.015**	**0.002**	**0.006**	**0.001**
Leukocyte concentration (10^6^/mL)	0.0	0.25 (0.25-0.25)	0.5 (0.5-0.5)	0.75 (0.75-0.75)	1.25 (1.0-1.93)				**0.001**

^a^Mann-Whitney t test

Category A: >0 to <0.25 × 10^6^/mL; category B: >0.25 to <0.5 × 10^6^/mL; category C: >0.5 to <0.75 × 10^6^/mL; category D: >0.75 to <1 × 10^6^/mL; category E: >1 × 10^6^/mL. Bold denotes values that differ significantly from category A. Data are given in medians and interquartile ranges.

**Figure 1:**
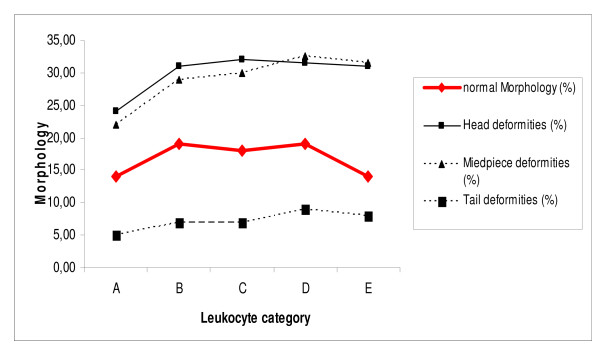
**Morphology (%) according to leukocyte concentration: category A: 0 to <0.25 × 10^6^/mL; category B: >0.25 to <0.5 × 10^6^/mL; category C: >0.5 to <0.75 × 10^6^/mL; category D: >0.75 to <1 × 10^6^/mL; category E: >1 × 10^6^/mL**. Data are given in medians; for detailed statistics see Table 1 and Results sections.

Median sperm concentration was significantly lower in category A samples than in category B, C, D and E samples. The highest sperm concentration was observed in category B samples (Table 1 and Fig. 1).

**Figure 2:**
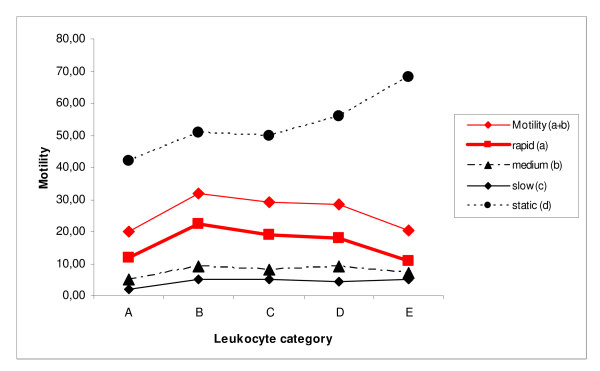
**Motility (%) according to leukocyte concentration: category A: 0 to <0.25 × 10^6^/mL; category B: >0.25 to <0.5 × 10^6^/mL; category C: >0.5 to <0.75 × 10^6^/mL; category D: >0.75 to <1 × 10^6^/mL; category E: >1 × 10^6^/mL**. Data are given in medians; for detailed statistics see Table 1 and Results sections.

The percentage of sperm with normal morphology was significantly higher in category B, C and D samples than in category A samples; percentages in category E samples were similar to those in category A samples (Table 1 and Fig. 1). Head, midpiece and tail deformities were significantly lower in category A samples than in samples from all four other categories (B, C, D, E). Motility grades a and a+b (progressive motility) showed profiles similar to that of normal morphology (Figs 1 and 2).

Using the Spearman Rho correlation for the overall data, statistically significant correlations were seen between leukocyte concentrations and motility grade b (*r *= 0.100, *P *= 0.013), grade c (*r *= 0.145, *P *= 0.001) and grade d (*r *= 0.192, *P *= 0.001), and between leukocyte concentrations and normal morphology (*r *= 0.113, *P *= 0.005), head deformities (*r *= 0.216, *P *= 0.001), midpiece deformities (*r *= 0.214, *P *= 0.001) and tail deformities (*r *= 0.162, *P *= 0.001).

Because of the distribution of results according to leukocyte category, a linear regression analysis was also calculated. In this analysis, significant correlations between leukocyte categories and head deformities (*P *= 0.012), midpiece deformities (*P *= 0.008) and grade d motility (*P *= 0.001) were found (see Figures 1 and 2).

## Discussion

The results of the current study are in agreement with those of both Aziz et al. and Ziyyat et al. mentioned previously [13,14]. In line with the results of Ziyyat et al., this study showed an increase in normal morphology and progressive motility in semen samples with leukocyte concentrations ranging from 0-1.0 × 10^6^/mL, but a decrease in each parameter at a leukocyte threshold >1 × 10^6^/mL (Figs 1 and 2). In contrast (and in line with the results of Aziz et al.), all types of sperm deformities increased progressively with increasing leukocyte counts. Thus, these results can be interpreted to imply that leukocytes might have both positive and negative effects on specific seminal parameters, which may be concentration dependent.

However, it has to be stated that we investigated leukocytes after Endtz staining and according to the WHO laboratory manual 1999 [1,16]. Therefore leukocytes represent just polymorphophonuclear granulocytes.

How can these contradictory effects be explained? The relationship between leukocytes and semen parameters appears to be highly complex, and may involve a number of factors, such as proinflammatory cytokines [17] and/or ROS. ROS, in particular, have featured widely in the recent literature as a possible factor in the association between leukocytes and semen parameters, and will form the focus of our discussion. Although the current study evaluated the effect of leukocyte concentration rather than ROS levels, leukocytes have been described in the literature as the primary source of ROS, and so could be viewed as a surrogate marker for ROS [12].

As has been mentioned, the observed negative effects of leukocytospermia on semen quality have been attributed to the presence of harmful ROS [9-11]. ROS are produced by leukocytes even at leukocyte concentrations <1 × 10^6^/mL, prompting suggestions that the leukocyte concentration used by the WHO to define leukocytospermia (>1 × 10^6^/mL) should be lowered [11,12]. However, ROS also have a physiological function in cell signalling and have been shown to induce sperm capacitation, hyperactivation, and the acrosome reaction [18,19]. Though the production of ROS by spermatozoa has also been reported [11], it remains to be determined whether this intrinsic ROS can have a detrimental effect on semen parameters. Henkel et al. found leukocytes to correlate more with extrinsic ROS production than with intrinsic ROS production [11].

Thus, the effects of ROS appear to be twofold and, hence, any effect on spermatozoa by leukocytes could also be double-edged.

So, why would it appear that leukocytes at concentrations of >0-1 × 10^6^/mL improve some aspects of semen quality, despite the fact that high ROS levels could be produced even at these fairly low and normal leukocyte concentrations [12]? The explanation may be that ROS have a very short half-life, and are generated constantly in the cell. However, seminal plasma has a large number of antioxidant defence mechanisms. This allows for the fact that any negative impact of ROS can be readily reduced by scavenger mechanisms [20]. Thus, the effect of ROS is dependent on the balance between oxidant and antioxidant activity, and ROS will only have negative effects once they exceed a specific threshold [10].

In recognition of this high seminal antioxidant capacity, Sharma, Agarwal and colleagues [10,12,21] suggested including the total antioxidant capacity (TAC) of seminal plasma to the measurement of ROS in the evaluation of male infertility [20]. Consequently, this group established that if the antioxidant capacity was sufficiently high, ROS did not harm spermatozoa, despite the presence of excessively high ROS levels. This phenomenon may also be relevant at leukocyte concentrations ranging from 0 to 1 × 10^6^/mL. In the study by Sharma et al. [12], the ROS concentration in the group with leukocyte concentration 0-1 × 10^6^/mL was low (2.62 ± 0.17 counted photons per minute) but the TAC score was significantly higher (*P *< 0.001) than that in the group with leukocytospermia >1 × 10^6^/mL. These findings clearly show that despite low numbers of leukocytes being able to produce ROS, the amount of cell damage is not always a consequence of ROS production, but is also dependent on the TAC.

One limitation of our study is that this was retrospective. However, the findings of a recent prospective study, published by Mafhouz et al. (2008), are also in agreement with a dual effect of ROS and seminal leukocytes [22]. The authors showed not only a differential shift between intracellular ROS (H_2_O_2_, O_2_^-^) in mature, immature and neat sperm fractions, but also that H_2_O_2 _exposure lowered the percentage of apoptosis in mature versus neat sperm; ROS levels in mature and immature fractions were similar. The authors concluded that ROS were not associated with an increase in apoptosis in immature and mature sperm fractions, probably because of adapting mechanisms for scavenging in the spermatozoa.

The finding that high intracellular H_2_O_2 _levels did not increase the level of dead sperm in the mature sperm fraction is supported by the results of the current study. In our study, normal morphology and motility (grades a+b) increased in semen samples with leukocytes concentrations 0-1 × 10^6^/mL, indicating that mature spermatozoa were responsible for these findings (assuming that normal morphology and motility grade "a" represent mature spermatozoa). Thus, normal spermatozoa may have the ability to compensate for a certain concentration of leukocytes. However, the specific pathway by which leukocytes may influence morphology cannot be clarified by the findings of this study.

## Conclusions

To summarize, the main findings of this study were that the percentages of sperm with normal morphology and progressive motility were higher in semen samples with a leukocyte concentration between 0 and 1 × 10^6^/mL than in samples with a leukocyte concentration >1 × 10^6^/mL. Although the relationship between leukocytes and semen parameters is a complex one, and may involve many different factors, these results indicate that leukocytes may have a positive effect on some semen parameters, which may be concentration dependent, and that the threshold for the concentration of leukocytes that might harm spermatozoa corresponds to the current WHO-defined level of >1 × 10^6^/mL [1].

## Competing interests

The authors declare that they have no competing interests.

## Authors' contributions

JL substantial contributions to concept, design, methodology, acquisition of data, statistics, interpreting the data and writing of the study. AA and RM substantial contribution to the analysis and interpretation of the data, drafting the manuscript and final approval. SP substantial contribution to the analysis of the data, revising the article critically for important intellectual content and final approval. GS substantial contribution to the acquisition of the data, statistics, interpreting the data and revising the manuscript critically.
